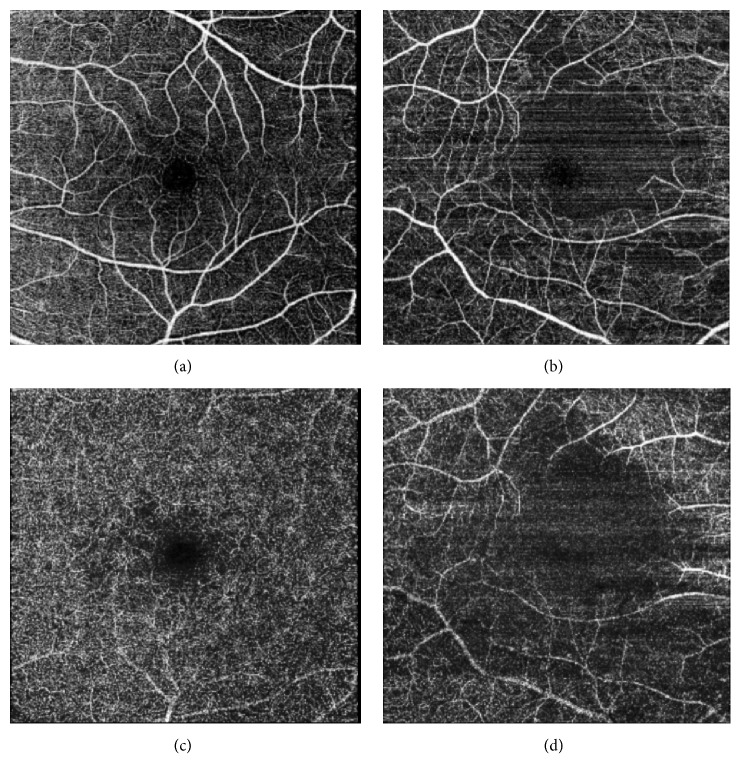# Optical Coherence Tomography Angiography of Purtscher Retinopathy after Severe Traffic Accident in 16-Year-Old Boy

**DOI:** 10.1155/2018/4318354

**Published:** 2018-03-01

**Authors:** Hassan Hamoudi, Marie Krogh Nielsen, Torben Lykke Sørensen

**Affiliations:** ^1^Clinical Eye Research Unit, Department of Ophthalmology, Zealand University Hospital, Roskilde, Denmark; ^2^Faculty of Health and Medical Sciences, University of Copenhagen, Copenhagen, Denmark

## Abstract

**Purpose:**

To describe optical coherence tomography (OCT) angiography (OCTA) in a case of Purtscher retinopathy.

**Methods:**

A 16-year-old male underwent ophthalmological examination including color fundus photography, spectral domain OCT, OCTA, and microperimetry. Examination was performed 10 days, 1 month, and 6 months after the trauma. Diagnosis was based on the characteristic clinical presentation.

**Patients:**

A single patient case.

**Results:**

Only the right eye was affected, and all examinations of the left eye were normal. The visual acuity of the right eye was 0.03 (Snellen equivalent) at 10 days and at one month, improving to 0.16 at 6 months. The imaging confirmed the findings of Purtscher retinopathy with ischemic whitening of the retina and retinal hemorrhages and thickened inner retina on OCT. Microperimetry showed reduced sensitivity in the central macula of the right eye. OCTA revealed nonperfusion in both the superficial and the deep retinal capillary plexus of the right eye.

**Conclusion:**

The OCTA in traumatic Purtscher retinopathy following traffic accident showed nonperfusion in both the superficial and the deep capillary plexus of the retina. OCTA is a valuable noninvasive diagnostic examination in Purtscher retinopathy, and fluorescein angiography became redundant in this case.

## 1. Introduction

Purtscher retinopathy is an extremely rare condition with an estimated incidence of 0.24 per million per year. It is a condition associated with numerous forms of trauma, including cranial trauma and thoracic compression. It is an occlusive microvasculopathy, and the clinical presentation includes loss of vision of varying severity, occurring hours to days after the trauma. The funduscopic findings include whitening of the retina, multiple cotton wool spots, and bleeding of different sizes. The retinal changes are explained by acute ischemia [1, 2].

In this case report we describe a patient with Purtscher retinopathy, examined with fundus photography, spectral domain optical coherence tomography (OCT) using Heidelberg Spectralis (Heidelberg Engineering, Germany), OCT-angiography (OCTA) (Topcon, Japan), and microperimetry using Microperimeter MP-3 (Nidek Co., Ltd., Japan). The novelty in this paper is the description of Purtscher retinopathy on OCTA.

## 2. Case Presentation

A 16-year-old boy was involved in a car accident as a front seat passenger. The patient had no previous or current medical history. In the initial phase he was hospitalized in the intensive care unit because of multiple injuries. He suffered from bleeding in the abdominal cavity, pneumothorax, and lesions of the spleen but experienced no direct head trauma or loss of consciousness during the accident. There were no signs of pancreatitis. He underwent abdominal surgery with laparotomy and tube thoracostomy. After a couple of days his general condition was stabilized and he was transferred to the Pediatric Department at our hospital. Ten days after the trauma he complained about blurred vision on the right eye, and was therefore referred to the Department of Ophthalmology.

**Figure 1:**
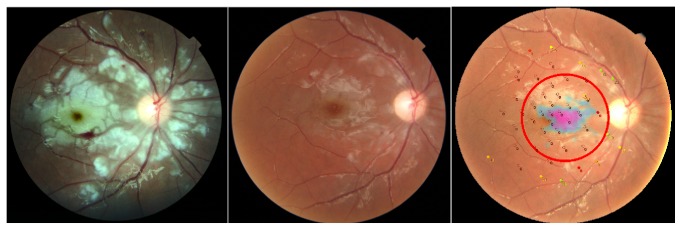


His presenting corrected visual acuity was 0.03 (Snellen equivalent) on the right eye and 1.0 on the left eye and was unchanged at one-month examination. Six months later, the visual acuity improved to 0.16 on the right eye and was still normal on the left eye. Anterior segment findings and intraocular pressure were normal. Ophthalmoscopy and fundus photo revealed an ischemic white posterior pole with cotton wool spots and intraretinal hemorrhages mainly in the macula and nasally to the optic disc. The white lesions and bleeding decreased already at the one-month visit (Figure 1), and after 6 months, the white lesions were almost resolved.

**Figure 2:**
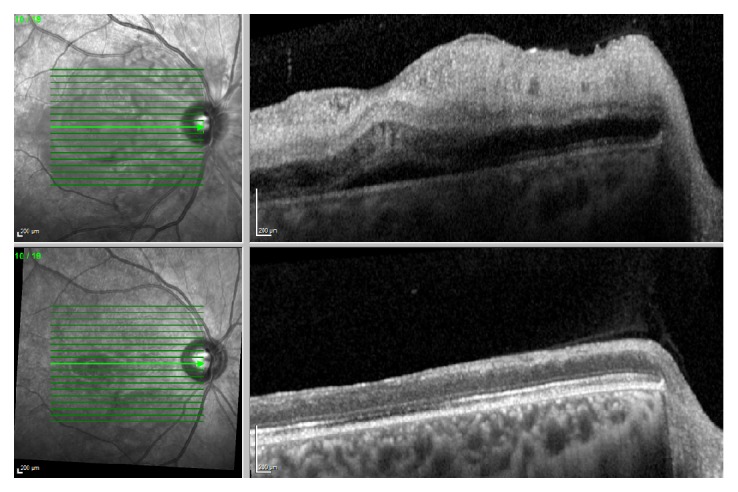


OCT showed hyperreflective and thickened inner retinal layers, a sign of ischemia in the inner retinal circulation. (Figure 2). At follow-up visits the edema decreased significantly on OCT with disruption of the inner retinal layers but also seemingly disrupted ellipsoid zone. In addition, the thickness of the retina was reduced, from 427 microns at onset to 207 microns at 6 months. A manual segmentation of the OCT layers was conducted in order to ensure correct layer identification and thus thickness calculation. Testing of the central visual field by microperimetry showed a central scotoma with decreased sensitivity in the fovea (Figure 1).

OCTA (Figure 3) revealed extensive nonperfusion in the macular area in both the superficial (Figure 3(b)) and the deep capillary plexus of the right eye (Figure 3(d)). OCTA of the left eye was with normal capillary plexus and normal foveal avascular zone (Figures 3(a) and 3(c)).

## 3. Discussion

Our patient had lower sensitivity in the fovea, which we found to correlate morphologically with the subfoveal atrophy found on OCT. The novelty in this case report is the description of Purtscher retinopathy on OCTA where we found nonperfusion in both the superficial and the deep capillary plexus in the macular region. OCTA allows a fast and noninvasive assessment of the retinal vascular structures and can detect vascular abnormalities without the need of fluorescein angiography [3].

Purtscher retinopathy is a rare condition that was first described by Otmar Purtscher in 1910 with findings of multiple retinal white lesions and superficial retinal hemorrhages. This was in a patient with head trauma, and the condition has since been described in various types of trauma, including seatbelt and airbag pressure, malar bone fracture, and chest trauma [4]. A similar retinal presentation has also been seen in patients without trauma but with a variety of conditions including acute pancreatitis, systemic lupus erythematosus, renal failure, and lymphoproliferative disorders. The condition is called Purtscher-like retinopathy because of the comparable clinical presentation but different etiological association.

The diagnosis of Purtscher retinopathy is made on the clinical presentation and the patient's history. The symptoms can be unilateral or bilateral and usually with immediate decrease of visual acuity. The characteristic findings are the Purtscher flecken, which are multiple cotton wool spots of varying sizes. The condition must be differentiated from other ocular disorders that may have common clinical features, for example, commotion retina, Terson's syndrome, shaken baby syndrome, and Valsalva retinopathy [5].

In our case the condition was unilateral, but the onset of the decreased vision is uncertain because of the severe general condition of the patient. The ischemia involving the inner retinal layers during the acute phase which we describe in our patient has previously been reported [6]. It is more difficult to identify the involvement of the outer retina in the acute phase because of the difficulty of its visualization. This is due to the thickened inner retina that appears to be interfering with the signal of the blood flow in the outer retina making the OCTA image not optimal. However, there are reports on photoreceptor disruption with loss of photoreceptor segments in the acute phase, recognized by multifocal electroretinography [7]. In other retinal conditions the finding of an alteration of the interface line between the inner and outer segments is an indication of suffering photoreceptors [8], and this may also be true in Purtscher retinopathy. The involvement of the photoreceptors may explain the visual field abnormalities found in this patient. The microperimetry provides the functional aspect of the morphological changes found on OCT with fundus-controlled testing allowing a precise retinal location [9].

The pathogenesis is still not completely clear, but some hypothesis exist. An increase in the thoracic pressure leads to a reflux in the venous system leading to endothelial damage. This results in incompetence of the microvascular circulation and subsequent occlusion and ischemia [5]. Another hypothesis suggests that the ischemia is the result of an emboli. Both air and fat emboli have been described due to trauma, and the source of the emboli may be thorax [10]. There is no standardized or recommended treatment, and the prognosis varies, some experience a recovery with a visual acuity of 6/12 Snellen or better; however, the prognosis is generally poor and the visual acuity may remain decreased particularly in case of foveal photoreceptor atrophy [5].

In conclusion, OCTA is valuable in the assessment of retinal vascular structures in Purtscher retinopathy and may replace invasive dye-based angiography. It also reveals ischemia at an earlier point.

## Figures and Tables

**Figure 3:**